# Gender gap in returns to publications

**DOI:** 10.1038/s41598-026-57252-4

**Published:** 2026-06-14

**Authors:** Piotr Śpiewanowski, Ivan Stetsyuk, Oleksandr Talavera

**Affiliations:** 1 Institute of Economics, Polish Academy of Sciences, Warsaw, Poland; 2 Zayed University, Abu Dhabi, United Arab Emirates; 3 University of Birmingham, Birmingham, UK

**Keywords:** Credit attribution gap, Discrimination, Gender pay gap, Research productivity, Women in science, Mathematics and computing, Psychology, Psychology

## Abstract

Gender equity in science depends not only on who participates in research, but also on how their work is rewarded. We examine gender gaps in monetary returns to research productivity, pay, and credit attribution using a novel dataset linking salary and publication records for professors at U.S. public universities. While our main contribution shows that there is no robust evidence of a significant gender gap in monetary returns to publications, we uncover substantial heterogeneity across disciplines and authorship structures. Women in medical and health sciences experience a negative gap in returns of 10.7% relative to men for equivalent research output. The unconditional gender pay gap averages 15.4%, with variation across disciplines, ranging from 5.5% in agricultural sciences to 20.9% in social sciences. It declines to 4.9% after controls. Finally, women earn higher returns to solo-authored work but lower returns to larger co-author networks than men.

## Introduction

Extensive literature documents persistent gender pay gaps across various industries^1–3^. However, researchers still do not know the size of the gender gaps in career outcomes (i.e., pay and promotion) attributable to differences in performance^4^. This gap persists in part because objective measures of such an important determinant of compensation as productivity are often unavailable, particularly for highly qualified professionals. For instance, conventional stock-based or accounting-based performance metrics are either not “precise” evaluation measures or they are subject to manipulation^5–9^. Consequently, addressing the gender gap in returns to performance requires a setting with precise, objective, and manipulation-proof performance measures.

In this paper, we address the aforementioned challenges by employing a research design that leverages a setting with highly objective and verifiable performance metrics, thus overcoming many of the limitations identified in prior studies. Specifically, we examine gender pay gaps and the monetary returns to research productivity within academia—an ideal context where a significant portion of tenure-track faculty performance, namely research output, can be measured objectively. By linking academic achievements to salary trajectories over time, we identify the monetary returns to research output. This allows us to test for the existence of a gender gap in these returns, which we refer to as the “gender gap in returns to publications.” Furthermore, we examine whether the attribution of credit differs by gender, by comparing the returns to solo-authored and co-authored publications for men and women across major academic disciplines.

Our analysis is based on a novel panel dataset linking annual salary records with detailed publication histories for over 32,000 faculty members at leading U.S. public universities. This rich, longitudinal dataset enables us to account for time-varying covariates and persistent individual heterogeneity while precisely measuring research productivity and collaboration patterns at scale. To our knowledge, this is the first study to empirically assess gender differences in returns to research productivity.

Our paper contributes to the broader debate about gender gaps in labor market outcomes, focusing on high-skilled workers. Academia offers a valuable setting for this inquiry because research productivity is the primary determinant of tenure-track faculty compensation, far outweighing teaching or service activities^10^. While performance-based pay aims to link compensation to productivity, it can simultaneously fragment women’s labor-market position and produce uneven outcomes for gender equity^11^.

Previous work shows that women publish less across academic disciplines^12,13^, a gap linked to discriminatory practices in grant funding, manuscript reviewing, and hiring^14,15^, as well as to promotions^16^. These structural barriers raise the possibility that men and women may be compensated differently for equivalent academic output, an idea we test empirically. Additionally, more subtle mechanisms may contribute to gender disparities in returns. For example, women may negotiate less aggressively for pay^17–19^, or face higher social costs when they do^20,21^. Gender-based expectations, role orientations, prejudice, and biased feedback may further penalize women by shaping how their performance is evaluated^22–26^.

Research also suggests that women may be systematically disadvantaged in collaborative environments. Papers with female co-authors receive fewer citations^27^. Female scholars are cited less than male scholars^28^. Female economists face penalties in tenure evaluations for co-authored publications^29^. Women’s co-authorship patterns differ from men’s, especially within highly ranked departments and prestigious journals^30^. Women occupy fewer brokerage positions in scientific collaboration networks, limiting their access to high-impact opportunities^31^. These patterns may also extend to compensation, with women earning less than men for co-authored research, another hypothesis we seek to test.

Recent evidence shows that gender pay disparities in academia arise not only from differences in discipline, rank, and experience but also from how compensation is structured^32^. Our study shifts attention from salary components to the returns to academic productivity itself. By linking detailed productivity records to annual compensation trajectories and distinguishing between solo- and co-authored research, we assess whether men and women receive comparable monetary rewards for equivalent scholarly output and whether credit attribution mechanisms embedded in collaborative work contribute to gender differences in performance-based compensation.

The main findings of this study can be summarized as follows. First, we find no consistent evidence of a gender gap in returns to publications among tenured and tenure-track faculty. On average, a 10% increase in research output is associated with a 1.65% increase in salary, with no significant differences between men and women. Thus, male and female faculty are compensated similarly for equivalent levels of research performance, whether measured by publication count or by various indicators of publication quality.

Second, we show that women earn less than men overall across all major academic disciplines. Much of this pay gap is explained by observable differences, not only in research productivity, but also in experience and academic rank, where female faculty consistently underperform their male peers at every career stage. After controlling for these factors, the gender pay gap declines from 15.4% (unconditional) to 4.9%, indicating that while most of the disparity is explained, a substantial residual gap remains. These findings are consistent with earlier evidence of gender disparities in academic compensation^33–36^ and align with broader trends in high-skilled labor markets^1^.

Third, we uncover a distinct gender gap in credit attribution related to collaborative research. While male faculty receive similar compensation for solo- and co-authored publications, after controlling for the number of co-authors, female faculty experience a marked asymmetry. Solo-authored research by women earns higher salary returns than comparable work by men, and women face a penalty as their co-author networks expand. The returns to co-authored publications themselves are statistically similar across genders in our preferred specification. Moreover, we find that men benefit from larger co-author networks, with corresponding increases in salary. In contrast, women do not benefit comparably from expanding their collaborative networks. These patterns suggest that female contributions to joint research are systematically undervalued, both within individual projects and across broader research partnerships. This attribution gap aligns with earlier findings that women are less likely to be recognized for joint work, particularly in male-dominated disciplines^29,37,38^.

Our findings are consistent with a broad literature documenting institutional and behavioral mechanisms that may sustain gender inequality. Women are less likely to negotiate for higher salaries^17,19^ and more likely to face penalties when they do^21^. They also receive more critical evaluations and are subject to ambiguous performance standards, particularly in male-dominated environments^22,23,26^. Broader labor market dynamics, such as statistical discrimination, career interruptions due to family responsibilities, and differences in risk preferences or competitiveness, may further reinforce these patterns^39–46^.

## Data and methods

### Data sources

We construct our analysis sample by selecting leading public universities in the United States for which reliable salary and publication data are available. Initially, we identify 116 institutions from those listed in the 2020 QS World University Rankings and the Wall Street Journal/Times Higher Education public university rankings. We obtain academic salary data for the period 2017–2019 from the U.S. Public Employee Salary and Pension Records, compiled by American Transparency, and we collect publication records from Scopus. The salary figure reflects total annual compensation as disclosed in the public payroll records, combining base salary with supplemental payments such as summer salary, administrative stipends, and clinical compensation, with composition varying across individuals and institutions (see Appendix A). Due to incomplete or inconsistent payroll data at some institutions, we refine our final sample to include 69 universities that provide comprehensive and consistently matchable data throughout the three-year period. These institutions are research-intensive and may operate under different compensation structures than private or teaching-oriented institutions.

To accurately link individual academics between payroll and publication datasets, we standardize names by removing special characters and converting text to lowercase. Additionally, we account for all known name variants associated with each Scopus author ID. To ensure matching accuracy and prevent misidentification, we adopt a conservative disambiguation strategy: records involving ambiguous name-to-Scopus ID relationships (such as a single name corresponding to multiple Scopus IDs or vice versa) are excluded. For authors successfully matched, we extract their entire publication histories available in Scopus, extending beyond the 2017–2019 window.

Our final dataset comprises 76,173 matched observations at 69 U.S. public universities, representing 32,694 distinct university employees and 6,790 singleton observations. Since gender is not consistently reported in payroll records, we infer gender using the Namsor API, which estimates gender based on individual names. We exclude observations with low-confidence gender classification (probability score below 0.25). The main results are robust to a stricter confidence criterion; see Appendix C.9 for the regression results and a discussion of the distribution of name-confidence scores. Complete details regarding data sources, selection criteria, and the matching methodology are presented in Appendix A.

### Measures of productivity

In the next stage of our analysis, we merge individual publication records with the SCImago Journal Rank (SJR) database, which provides journal-level quality indicators, including the H-index for all Scopus-indexed journals and the SJR score. The SJR score is a size-independent measure of journal quality, computed using an algorithm akin to PageRank that weights citations by both their number and the prestige of the citing sources, with greater weight placed on citations from closely related journals. The database also classifies journals into academic disciplines. We utilize these indicators to construct measures of individual research performance.

For each author and each year, we construct cumulative productivity as the SJR-weighted sum of their publications up to that year, with each publication credited at $1/\sqrt{n}$, where *n* is the number of co-authors. Fractional counting of co-authored publications is standard practice in research evaluation and is used in national publication-based funding systems such as those in Norway, Flanders, and Poland^47,48^. The $1/\sqrt{n}$ (“modified fractional”) scheme is intermediate between full credit and equal credit (1/*n*). As a robustness check on the credit-attribution decomposition in Table 5, we re-estimate it under full credit, 1/*n*, and the baseline $1/\sqrt{n}$ in Appendix C.4. Mean co-author team size is similar by gender (7.9 vs. 7.8 co-authors per paper for men and women), and the shares of solo-, first-, and last-authored work by gender and broad field are reported in Appendix C.5 , Table C5.

Throughout the paper, we use *broad field* to refer to the six SJR-derived disciplinary categories (Agricultural Sciences, Engineering and Technology, Humanities, Medical and Health Sciences, Natural Sciences, Social Sciences), assigned at the author level using the modal SJR broad category across the author’s full publication record, with ties broken at random among the dominant categories. *Narrow field* refers to the approximately 300 SJR sub-disciplinary categories, used as fixed effects in the main regressions. *Experience* is a continuous variable measured as years since the first Scopus-recorded publication; *experience-year bins* are five-year fixed-effect bands used in the regression controls. The salary–publication match is performed at the person level (one Scopus ID matched to one payroll record), yielding up to three person-year observations per matched individual; further detail on the matching procedure is in Appendix A.

As a further robustness check we construct alternative productivity measures based on unweighted publication counts and on the journal-level H-index.

### Data summary

**Fig. 1:**
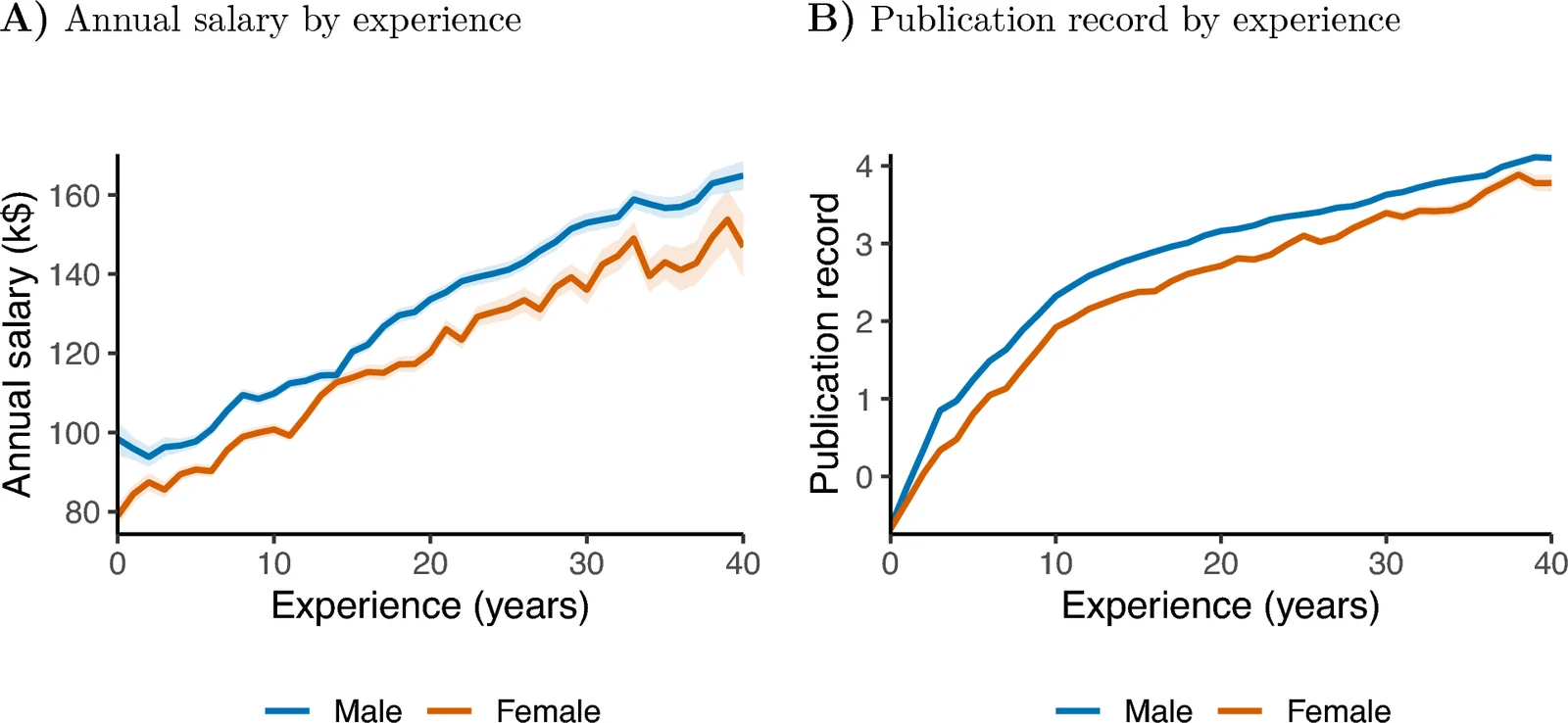
Gender gap in salaries and publication record by experience. (**A**) Annual salary (k$) by years since first publication. (**B**) Publication record, measured as the natural logarithm of cumulative journal quality (SJR), with each publication weighted by the inverse of the square root of the number of authors. Shaded areas represent ±1 standard error around the estimated mean values for each gender.

In Fig. 1, we present a visual summary of salary distributions and our primary measure of research productivity across different experience levels. We show that females consistently receive lower salaries and publish less compared to men across all experience levels. Experience is defined as the number of years since an individual’s first publication; if a faculty member is hired one year or more before their first publication, the experience measure can be negative. Specifically, Panel A of Fig. 1 demonstrates that faculty salaries rise smoothly with experience for both genders, yet a persistent unconditional gender pay gap remains evident at every career stage.

Panel B of Fig. 1 illustrates the trajectory of research productivity, measured as the natural logarithm of cumulative SJR scores, with each publication weighted by the square root of the number of co-authors. The productivity curve rises most steeply in the initial years of an academic career, suggesting an increase in annual research productivity shortly after a researcher’s first publication. This trend is consistent with intensified early-career research activities, potentially driven by tenure-track incentives. Over time, as researchers gain experience, the curve gradually flattens, indicating a slowdown in the growth rate of cumulative research output. These findings align with previous studies that document higher male productivity across various industries^4,12,49,50^. Our findings corroborate prior evidence on the persistence of this gender productivity gap across career stages.

Table 1 compares summary statistics of the main variables for male and female faculty members. The average annual salary for male faculty is $151,405, which exceeds the female average salary of $127,346 by approximately 19%. Male faculty also demonstrate notably higher productivity measured by either publication count or SJR score, aligning with prior findings that women publish less^51^. Specifically, the mean number of publications for male faculty is 46.2, which is 74% higher than the mean number of publications by their female counterparts. Women represent 32% of faculty members in our sample. The median salary ($121,600) is lower than the mean salary ($143,617), indicating a right-skewed salary distribution influenced by a small number of very high-earning faculty. A pronounced disparity is also evident in research productivity metrics, including both publication counts and cumulative SJR scores; full-sample descriptive statistics are reported in Appendix B, Table B1.

**Table 1:** Descriptive statistics by gender.

Variable	Female		Male		Difference in means	T-Statistic of the difference
	Mean	Std. Dev.	Mean	Std. Dev.		
Salary (in $)	127346.10	71462.25	151405.38	94564.12	− 24059.28	− 38.97
Publication count	26.64	35.13	46.24	57.34	− 19.60	− 58.05
Log cumulative SJR score	23.47	37.99	45.17	67.10	− 21.70	− 56.77
Experience (in years)	16.50	10.17	21.38	11.72	− 4.89	− 58.95
Assistant Professor	0.29	0.46	0.20	0.40	0.09	26.17
Associate Professor	0.34	0.47	0.27	0.44	0.07	19.12
Full Professor	0.36	0.48	0.52	0.50	− 0.16	− 42.33
Broad field						
Agricultural sciences	0.07	0.25	0.07	0.26	− 0.01	− 2.97
Engineering and technology	0.03	0.18	0.08	0.27	− 0.05	− 29.62
Humanities	0.05	0.22	0.03	0.17	0.02	11.73
Medical and health Sciences	0.36	0.48	0.28	0.45	0.09	23.66
Natural sciences	0.17	0.38	0.33	0.47	− 0.15	− 48.87
Social sciences	0.31	0.46	0.21	0.41	0.10	30.22

N = 76,173 observations

Additionally, the average log cumulative SJR score for males exceeds that for females by 93%. These productivity differences could partially reflect the 30% higher average experience level among male professors. Nonetheless, our findings corroborate trends of increasing research productivity among female researchers in the U.S., as documented by Frietsch et al.^52^ Appendix B, Table B2 highlights cross-disciplinary heterogeneity in academic careers, including large differences in salaries, research output, and experience across fields, as well as sharp variation in female representation by discipline.

We further examine academic ranks, motivated by prior evidence suggesting that the increasing proportion of women in entry-level positions contributes to lower average salaries^53^. Faculty rank also accounts for a significant gap in base pay^32^. Our analysis shows that the proportions of women are higher in junior academic ranks (Assistant and Associate Professor), but their representation markedly declines at senior ranks. Specifically, Table 1 reveals that 52% of male faculty members hold Full Professor positions, compared to only 36% of females. These findings align with the previously documented “leaky pipeline” phenomenon, where female representation declines at higher academic ranks^54^. This pattern is often linked to the impact of family responsibilities and substantial parenthood penalties in academia, particularly among women. Career interruptions associated with childbirth and caregiving can reduce cumulative publications, slow promotion, affect grant acquisition and collaboration opportunities, and alter long-run salary trajectories (e.g.,^41,42,55^). These effects may operate not only through observed publication outcomes, but also through less visible channels such as professional network formation, committee participation, and institutional visibility.

Regarding representation across disciplines, female faculty are more prevalent in the humanities, medical and health sciences, and social sciences. Notably, 36% of female faculty members are in medical and health sciences, whereas only 3% work in engineering and technology, consistent with prior evidence^14^. In contrast, 33% of male faculty members are in natural sciences, and only 3% are in humanities. As shown in Appendix B, Table B3, substantial gender disparities persist across all scientific fields: women consistently earn lower salaries, publish less, and accumulate lower SJR-weighted research impact than men, even after accounting for variation within disciplines. These gaps are especially pronounced in Engineering and Technology, Medical and Health Sciences, and the Social Sciences, underscoring systemic inequalities in academic careers. Such substantial gender disparities across academic fields point to the role of structural factors shaping faculty career trajectories and choices, and are consistent with prior evidence that women receive fewer awards and less prestige for their research than men^56,57^.

This distribution across disciplines is also important for interpreting the later field-level results on returns to research productivity. Medical and health sciences represent the single largest field for female faculty in our sample, accounting for 36% of women compared with 28% of men. As we show in Appendix C1, this field is also the only broad field in which women experience a statistically significant negative gap in returns to publications, earning approximately 10.7% lower salary returns than male colleagues for equivalent research output. The concentration of women in this field may therefore contribute disproportionately to aggregate gender disparities in academia, underscoring the importance of discipline-specific analyses.

### Methods

Our objective is to precisely measure the size of the gender gap, controlling for individual time-invariant characteristics and academic achievements (research productivity), and to estimate gender differences in returns to research productivity. To fully exploit the unique properties of our sample, which allow us to account for persistent individual heterogeneity while also estimating differences along the time-invariant dimension of gender, we employ the Correlated Random Effects (CRE) approach^58–60^.

In contrast to the standard fixed-effects models, the CRE model also enables the estimation of coefficients for time-invariant variables such as gender, which is central to our research question. Identification of the coefficient on a time-invariant regressor in the CRE framework comes from cross-individual variation conditional on the within-person means of the time-varying covariates, that is, from individuals who differ in gender but are otherwise comparable on time-averaged observable characteristics. Interactions between time-invariant variables (e.g., Female) and time-varying ones (e.g., Productivity) are identified from the extent to which the productivity trend over the panel differs by gender, conditional on individual means^61^. The CRE approach controls for the mean of time-varying independent variables, making its estimates for these variables equivalent to those of a within-estimator. We perform a standard CRE estimation of the following model:

1 $$\begin{aligned} y_{i,t}= \beta _0 + \beta _1 Female_i + \beta _2 \left( x_{i,t} - \overline{x_{i}}\right) ' + \beta _3 \overline{x_{i}}' + \lambda _t + \mu _i + \epsilon _{i,t} \end{aligned}$$

where the main dependent variable ($y_{i,t}$) is the logarithm of the salary (in USD) of individual *i* in period *t*. The primary independent variable ($x_{i,t}$) is *Productivity*, measured as the natural logarithm of the cumulative SJR score of all publications authored by individual *i* up to time *t*, where each publication is weighted by the inverse of the square root of its number of authors (e.g., a three-authored publication contributes its SJR score divided by $\sqrt{3}$ to the cumulative total). To examine whether there is a gap in the returns to publications, we interact gender and productivity ($Female_i \times Productivity_{i,t}$) in selected specifications. The key time-invariant variable of interest is $Female_i$, a binary indicator equal to 1 for female scholars and 0 otherwise; its coefficient captures the average gender pay gap not explained by the included covariates. We include year fixed effects $\lambda _t$ and an individual-specific component $\mu _i$, and we condition on individual means of the time-varying covariates as in the CRE approach. All specifications include year fixed effects and control for the employing university in period *t*.

Because the within component of the CRE estimator is identified from individuals observed in more than one year, singleton observations contribute to the cross-sectional component through their individual mean but not to the within-individual variation in time-varying covariates; Appendix C.7 confirms that excluding singletons leaves the main coefficients qualitatively unchanged. In addition, for most specifications we present results of standard pooled OLS regressions using the same set of variables as in the CRE regressions but without individual fixed effects. As a robustness check, we also estimate the CRE model within a matching or weighting framework to account for potential differences in the distribution of control variables across genders. We employ various matching and weighting procedures: first, we construct a cross-sectional sample from each individual’s first observed year and apply the matching or weighting method using the full set of dependent variables from the CRE model. We then re-estimate the CRE model, as outlined in Equation 1, weighting observations according to the previously obtained weights. Note that these weights remain fixed over time, which may introduce bias if panel attrition rates differ by gender and correlate with the dependent variable.

As a further robustness check, we repeat the matching and weighting procedures after also incorporating the dependent variable, the logarithm of the salary, from the first observed year into the algorithm, thus ensuring the sample is matched along that dimension as well. From the broad set of matching and weighting techniques, we focus on coarsened exact matching, nearest neighbor matching, and inverse probability weighting. Although different methods can yield different sample sizes^62^, our findings, as presented in the results section, remain qualitatively consistent across these approaches.

## Results

### Gender pay gap and gender gap in returns to publications

In this section, we present progressively richer specifications to examine both the magnitude of the gender pay gap and the gender gap in returns to publications. The early specifications estimate unconditional and conditional gender pay gaps, while the later models introduce the *Female* × *Productivity* interaction, which directly tests whether men and women receive different marginal returns to equivalent research output. This distinction is central to the paper, as the primary contribution of the analysis lies in examining gender differences in the returns to research productivity rather than salary differences alone.

We begin by examining the extent to which the gender pay gap is explained by differences in research productivity and other observable characteristics. Table 2 presents a series of salary regressions, progressing from pooled OLS specifications (Columns 1–4) to our preferred correlated random effects (CRE) models with individual fixed effects (Columns 5–6). In the baseline model in Column 1, which includes only year fixed effects, the unconditional gender pay gap is 15.4%. Controlling for research productivity using our author-adjusted SJR measure in Column 2 reduces this gap to 6.0% and increases the $R^2$ from 1.9 to 12.6%.

**Table 2:** Gender pay gap and gender gap in returns to publications.

	Pooled				CRE	
	(1)	(2)	(3)	(4)	(5)	(6)
Female	− 0.154***	− 0.060***	− 0.041***	− 0.017	− 0.062***	− 0.049***
	(0.006)	(0.006)	(0.005)	(0.012)	(0.005)	(0.012)
Productivity		0.138***	0.091***	0.094***	0.169***	0.165***
		(0.002)	(0.003)	(0.003)	(0.017)	(0.021)
Female × Productivity				− 0.009*		0.010
				(0.004)		(0.027)
Num. obs.	76,173	76,173	76,173	76,173	76,173	76,173
*R*^2^	0.019	0.126	0.405	0.405	0.858	0.858
FE: Year	X	X	X	X	X	X
FE: Experience-Year Bins			X	X	X	X
FE: University			X	X	X	X
FE: Narrow Field			X	X	X	X
FE: Scopus ID					X	X

^+^*p* < 0.1, *p < 0.05, **p < 0.01, ***p < 0.001. Standard errors clustered at the individual (Scopus ID) level. University-level clustering yields qualitatively similar results; see Appendix C.8

Columns 3 to 6 progressively add controls for university and narrow academic field (with a fixed effect for each of nearly 300 SJR sub-disciplinary categories), and for experience-year bins of five-year width. The CRE specifications in Columns 5 and 6 additionally include individual (Scopus ID) fixed effects, which absorb persistent researcher characteristics including largely time-invariant rank classifications over our short 2017–2019 panel. Standard errors in all main-text regression tables (Tables 2, 3, 4, and 5) are clustered at the individual (Scopus ID) level to account for serial correlation within authors over the three-year panel.

We use experience-year bins as our main career-stage control. Rank is closely correlated with experience in academic careers, and as Appendix C.6 shows, adding explicit rank indicators does not change the main coefficients. The same is true when we drop singletons or restrict the sample to the balanced panel of individuals observed in all three years (Appendix C.7), or when we change the clustering unit from the individual to the university (Appendix C.8).

Adding further controls for university fixed effects, and narrow academic field, with a fixed effect for each of nearly 300 identified subfields in Column 3, further reduces the gap to 4.1%, with the $R^2$ increasing to 40.5%. Column 4 introduces an interaction between gender and productivity, which is negative and statistically significant in the pooled setting, suggesting lower returns to research output for women. However, we caution against interpreting this as evidence of differential treatment, as the model does not yet account for unobserved individual heterogeneity.

Columns 5 and 6 of Table 2 present a more comprehensive CRE specification, which includes Scopus ID fixed effects to control for time-invariant individual characteristics. This approach allows us to isolate the average gender pay gap while accounting for persistent researcher-specific characteristics. However, we acknowledge that time-varying changes in teaching loads, service burdens, clinical duties, and administrative appointments are not directly observed and may still affect salary. If these relevant determinants of faculty compensation differ systematically by gender, their omission could bias the residual gap upward or downward.

The gender–productivity interaction becomes statistically insignificant in Column 6, indicating no detectable difference in the marginal returns to productivity between men and women once persistent researcher-specific characteristics are accounted for. In both CRE specifications, research output remains a strong predictor of salary, and the $R^2$ rises to 85.8%, reflecting the substantial explanatory power gained by including individual fixed effects.

Importantly, this aggregate interaction may conceal heterogeneity across publication structures. The productivity measure used in Table 2 combines solo- and co-authored research into a single index, such that positive and negative gender-specific components may offset one another in the aggregate specification. We return to this issue in “Gender gap in credit attribution” section, where we separately examine the returns to solo-authored work, co-authored work, and co-author network size.

Taken together, these results show that much of the gender pay gap is attributable to differences in research output and other observable factors. Importantly, we find no consistent evidence that women receive lower pay for equivalent research performance.

### Robustness checks

We assess the robustness of our main findings using alternative measures of scholarly productivity, including publication count, weighted publication count, SJR score, and a co-author-weighted H-index in Table 3. All specifications follow the same modeling strategy as Column 6 of Table 2, incorporating correlated random effects and individual fixed effects.

**Table 3:** Gender pay gap and various measures of productivity.

	Publication count	Publication count by # Authors	SJR	H Index by # Authors
	(1)	(2)	(3)	(4)
Female	− 0.057***	− 0.058***	− 0.049***	− 0.064**
	(0.015)	(0.014)	(0.012)	(0.020)
Productivity	0.202***	0.216***	0.152***	0.076***
	(0.021)	(0.023)	(0.018)	(0.013)
Female × Productivity	− 0.020	− 0.003	− 0.005	− 0.020
	(0.024)	(0.028)	(0.023)	(0.016)
Num. obs.	76,173	76,173	76,173	76,173
*R*^2^	0.859	0.858	0.858	0.858
FE: Experience− Year Bins	X	X	X	X
FE: University	X	X	X	X
FE: Scopus ID	X	X	X	X

^+^*p* < 0.1, *p < 0.05, **p < 0.01, ***p < 0.001. Standard errors clustered at the individual (Scopus ID) level. All regressions estimated using the CRE model

First, we find that the gender pay gap remains statistically significant across all specifications. The magnitude of the gap varies slightly with the productivity measure, ranging from 4.9% (using SJR) to 6.4% (using the weighted H-index), and the $R^2$ remains consistently high. Second, all specifications confirm positive returns to research output. Third, we find no strong evidence of a gender gap in returns to publications. The interaction term between gender and productivity is statistically insignificant across all four productivity measures. These results confirm that our main findings are robust to a variety of productivity measures: while women earn less overall, they receive comparable compensation to men for equivalent research output.

To provide additional evidence beyond the main text, several appendices present robustness analyses examining heterogeneity in the gender pay gap and returns to research productivity. Appendix C1 shows that gender wage disparities differ meaningfully across academic fields, ranging from 5.5% in agricultural sciences to more than 20% in social sciences when productivity is not controlled for. Introducing productivity narrows the gap in some areas, eliminating it in agricultural sciences and humanities. Adding individual fixed effects substantially increases model fit and reduces the economic size of the gap. Field-specific interactions further show that women in medical and health sciences receive 10.7% lower returns to publications than men with identical output, explaining the absence of aggregate-level differences.

We examine heterogeneity across rank, experience, and productivity in Appendix C2. Women in junior ranks face the largest wage penalties, with adjusted gaps of 7.7% for assistant and 4.7% for associate professors, while the gap narrows for senior faculty and disappears after roughly 35 years of experience. In terms of publication returns, penalties are concentrated among the least productive half of researchers, even after controlling for detailed sub-discipline and individual characteristics. Salary returns to productivity rise during the first two decades of an academic career but turn negative at very long tenures.

We test whether delayed recognition of research output alters these conclusions by using lagged SJR scores in Appendix C3. Across one-, two-, and three-year lags, the results remain qualitatively unchanged: there is no evidence of a gender gap in returns to productivity. The productivity coefficient declines with longer lags, indicating that salary rewards for research impact accrue primarily in the first few years after publication.

**Table 4:** Robustness: matching and weighting.

	CE	NN	NNR	IPW
	(1)	(2)	(3)	(4)
Female	− 0.055***	− 0.078***	− 0.072***	− 0.056***
	(0.014)	(0.013)	(0.018)	(0.013)
Productivity	0.149***	0.146***	0.140**	0.145***
	(0.028)	(0.025)	(0.048)	(0.028)
Female × Productivity	− 0.013	− 0.004	0.009	− 0.005
	(0.031)	(0.030)	(0.048)	(0.031)
Num. obs.	72,167	49,230	40,934	76,089
*R*^2^	0.855	0.856	0.855	0.901
FE: Year	X	X	X	X
FE: University	X	X	X	X
FE: Scopus ID	X	X	X	X

^+^*p* < 0.1, *p < 0.05, **p < 0.01, ***p < 0.001. Standard errors clustered at the individual (Scopus ID) level. All regressions estimated using the CRE model on the matched or weighted sample

To minimize the influence of confounding factors, we employ four matching methods: coarsened exact (CE), nearest neighbor (NN), nearest neighbor with replacement (NNR), and inverse-probability weighting (IPW), reported in Table 4. The variables used for matching include experience, position, and faculty. The balance table for each matching procedure is reported in Appendix D, Table D1.

All matching estimators confirm the existence of a gender pay gap, which ranges from 5.5% under CE in Column 1 to 7.8% under NN in Column 2. Similarly, productivity consistently shows a significant and positive association with salary. Moreover, CE, NN, NNR, and IPW estimators all indicate that the gender gap in returns to publications is not statistically significant, as the interaction term remains insignificant. As shown in Appendix D, Table D2, a similar picture emerges once we include salary in the first sample year among the matching variables. The balance table for those matches is reported in Appendix D, Table D3. Overall, these exercises confirm the main results presented above.

### Gender gap in credit attribution

To explore how collaborative research structures shape compensation outcomes, we investigate the role of co-authorship in salary determination in Table 5. The specification uses the same fixed-effect set as Table 2. In Column 1, we find that both solo and collaborative research contributions are positively associated with faculty salaries. We also find a positive association between co-author network size and salary. Moreover, as Columns 2 and 4 show, while the returns to co-authored publications are relatively similar for male and female authors, the returns to solo-authored publications are higher for women. This effect is significant in the pooled specification (Column 2) and marginally significant (*p<0.10*) in the CRE specification with interactions (Column 4). This pattern is consistent with the idea that women face less bias when authorship credit is unambiguous^63^.

**Table 5:** Gender pay gap in returns to solo-authored work and co-author network size.

	Pooled	Pooled	CRE	CRE	Pooled	Pooled	CRE	CRE
	(1)	(2)	(3)	(4)	(5)	(6)	(7)	(8)
Female	− 0.042***	− 0.025*	− 0.062***	− 0.054***	− 0.041***	− 0.007	− 0.061***	− 0.058***
	(0.005)	(0.010)	(0.005)	(0.010)	(0.005)	(0.012)	(0.005)	(0.012)
Productivity (co-authored)	0.082***	0.085***	0.151***	0.148***				
	(0.003)	(0.003)	(0.015)	(0.018)				
Productivity (solo-authored)	0.021***	0.017***	0.004	− 0.018				
	(0.003)	(0.003)	(0.017)	(0.024)				
Female × Productivity (co-authored)		− 0.010**		0.009				
		(0.004)		(0.025)				
Female × Productivity (solo-authored)		0.015**		0.059^+^				
		(0.005)		(0.031)				
Productivity (total)					0.077***	0.077***	0.122***	0.093***
					(0.004)	(0.004)	(0.019)	(0.025)
Co-author count (log)					0.019***	0.022***	0.051***	0.075***
					(0.003)	(0.004)	(0.011)	(0.017)
Female × Productivity (total)						− 0.002		0.068^+^
						(0.006)		(0.036)
Female × Co-author count (log)						− 0.008		− 0.053*
						(0.005)		(0.021)
Num. obs.	76,173	76,173	76,173	76,173	76,173	76,173	76,173	76,173
*R*^2^	0.405	0.406	0.858	0.858	0.406	0.406	0.858	0.858
FE: Year	X	X	X	X	X	X	X	X
FE: Experience-Year Bins	X	X	X	X	X	X	X	X
FE: University	X	X	X	X	X	X	X	X
FE: Narrow Field	X	X			X	X		
FE: Scopus ID			X	X			X	X

^+^*p* < 0.1, *p < 0.05, **p < 0.01, ***p < 0.001. Standard errors clustered at the individual (Scopus ID) level

In Columns 5 through 8, we examine the returns to the size of the co-author network. We find that men’s salaries increase with the number of co-authors, even when controlling for productivity, potentially because co-author networks facilitate accurate estimation of potential productivity^64^. However, adding the interaction term between the female indicator and the logarithm of co-author count in Columns 6 and 8 reveals a negative association between co-authorship and female compensation, consistent with previous evidence that women may be penalized for co-authored publications^29,38^. The Female × Solo premium is robust to alternative co-author weighting schemes (Appendix C.4).

## Discussion

This study examines the gender pay gap and gender differences in returns to research productivity across academic disciplines, drawing on a rich dataset that merges salary information and publication records for faculty at U.S. public universities. It adds precision to the discourse on the gender pay gap in academia by comprehensively analyzing various metrics of research productivity and collaboration patterns across multiple disciplines. Our findings reveal an unconditional gender pay gap of 15.4%. It varies considerably across disciplines, from 5.5% in agricultural sciences to 20.9% in social sciences, indicating notable disparities in academic compensation practices. After accounting for key salary determinants, including research productivity, experience, academic rank, and specific academic fields, we estimate an average gender pay gap of 4.9%. This conditional gap remains statistically significant, pointing to persistent, albeit reduced, unexplained differences in pay. It may reflect a combination of discrimination, unobserved time-varying job responsibilities, salary-setting practices, and other factors not captured in our data.

We find no robust evidence of a significant gender gap in monetary returns to research productivity when estimating the models with precise individual-level data and relying on correlated random effects or matching estimators. Our estimates suggest that while there is a substantial unconditional pay gap, conditional on detailed measures of research productivity and individual characteristics, men and women receive broadly similar monetary returns to publications.

We uncover important differences regarding collaborative versus individual research in further analysis. Female faculty members receive significantly lower compensation for co-authored publications relative to male colleagues, indicating a specific gender penalty in the valuation of collaborative research. This penalty can depress career-long earnings even in the absence of a direct productivity-related pay gap. Conversely, solo-authored research by female faculty is consistently rewarded, at times even more positively than similar work authored by men.

Together, these results refine prior characterizations of the academic gender pay gap. On average, men and women receive broadly similar marginal returns to publications, so the residual conditional pay gap must reflect channels other than differential rewards for productivity. Beneath this aggregate, however, the structure of credit attribution differs by gender. Women earn higher returns to solo-authored work but face a penalty as their co-author networks expand, with the heterogeneous components largely offsetting each other in the aggregate.

By demonstrating that research productivity is a key determinant of compensation, we can reasonably infer that narrowing the productivity gap between men and women can mitigate the gender pay gap. At the same time, several data limitations should be acknowledged when interpreting the external validity of the results. The Female *×* Solo premium may partly reflect positive selection of women into solo-authorship on unobserved dimensions. In addition, the relatively short three-year panel limits within-individual variation, such that the estimates capture only changes in output observed during 2017–2019. Future research using richer longitudinal data should further investigate the sources of the productivity gap and the mechanisms constraining productivity, thereby helping academic institutions design policies that create a more level playing field for female faculty.

## Supplementary Information


Supplementary Information.


## Data Availability

The anonymized data and code are available from the corresponding author upon reasonable request.
