# Supplementary material for: Gender gap in returns to publications
Source: Sci Rep. 2026 Jun 14;16:27474. doi: 10.1038/s41598-026-57252-4 (PMC13534576; doi:10.1038/s41598-026-57252-4)
Supplement: Supplementary file 1 [file 41598_2026_57252_MOESM1_ESM.pdf]

Supplementary Material

for *Gender Gap in Returns to Publications*

Spiewanowski, Stetsyuk, and Talavera

A. Data Set

We utilize data from multiple sources to construct our analysis sample. First, we identify leading public universities in the United States. Specifically, we manually select 97 U.S. public universities from the list of 160 U.S. institutions that appear in the QS World University Rankings 2020 (QS).[1] We then supplement this list by including additional institutions that do not appear in the QS rankings but are featured in the Wall Street Journal/Times Higher Education U.S. public university rankings.[2] This process yields a total of 116 public universities for our study. For each university, we obtain salary data from the U.S. Public Employee Salary and Pension Records compiled by American Transparency (OpenTheBooks).[3]

The salary figure reported by OpenTheBooks corresponds to total annual compensation as disclosed in the public payroll records of each institution. This combines base salary with supplemental payments such as summer salary from grants, administrative stipends, and (in health sciences) clinical compensation. The exact composition varies across individuals and institutions. We acknowledge this measurement variation as a potential source of noise, particularly in the medical and health sciences, where clinical-supplement variation across universities may attenuate the estimated returns to research output.

Because salary data availability varies, we limit our analysis to the years 2017, 2018, and 2019, periods during which most universities report complete data. In some cases, salary data for campuses within the same university system are reported separately (e.g., University of Maryland, Baltimore County and University of Maryland, College Park), whereas others report data collectively at the system level. Accurate matching of salary data with research productivity data requires clear job title information. Institutions that do not provide job titles or provide only pension information without job titles further reduce our final sample to 69 universities. The database does not include information on federal academies for some states (e.g., Hawaii) and does not report information on several state public universities. Furthermore, for some institutions only pension data are available; we exclude such institutions from the sample.

For each institution in our sample, we manually identify the Scopus institution ID using Scopus Affiliations Search. Scopus is the largest abstract and citation database of peer-reviewed literature: scientific journals, books, and conference proceedings. Scopus Affiliation Search was accessed on 22/3/2021 at `https://www.scopus.com`. We manually screen search results to eliminate institutions registered outside the country or state where the institution is based (e.g., affiliated institutions abroad). We also manually exclude institutions that are erroneously displayed (e.g., if California State University is displayed among the results for University of California). Typically, institutions have multiple Scopus affiliation IDs for schools and departments, and we remove duplicates.

To identify individual authors affiliated with these institutions for 2017–2019, we implement an indirect method due to limitations with the Scopus API, identifying a total of 1,045,092 unique authors (Scopus IDs). For each author, we collect all name variations extracted from the variable "author names" appearing across their publications. In the next step, we match the payroll data and the institution-specific author lists separately for each institution. The match is based on the "Name" variable from the payroll database. Matching payroll data with author lists involves creating a standardized matching key by removing non-alphanumeric characters (including spaces) and converting names to lowercase. The newly created variable (Scopus name key) is subsequently

used as a matching key.

To ensure the matching process is successful, we must guarantee the uniqueness of the matching key (Scopus ID). Scopus collects data provided by journal publications. Depending on the name format required by the journal or due to inconsistency on the author's side, a single author may be linked to multiple name strings (e.g., James Smith and Jim Smith can both be the names for the same person). For matching, we use all name versions.

In our sample, we sometimes find multiple author IDs associated with a single name string. This can occur for two reasons. First, common names (e.g., John Smith) may be shared by multiple individuals. Second, errors in the Scopus data can split one author's publication record across multiple IDs. Because we cannot determine with certainty why multiple matches arise in each case, we exclude any name key–Scopus author ID pairs that appear more than once. For example, suppose we observe the following matches: Jim Smith (ID 01), James Smith (ID 01), and James Smith (ID 02). In such a case, we drop all observations with names Jim Smith and James Smith and with Scopus IDs 01 and 02. Next, we match all remaining unique author name key–Scopus author ID pairs for each institution with the payroll data. Finally, for all Scopus author IDs matched with the payroll data, we obtain the full publication record via the Scopus API.

This process leaves us with a dataset containing 143,131 observations, 63,132 unique matched individuals, and 15,412 singletons. We then assign gender to reported names using the Namsor API and exclude observations with imprecise gender assignment, defined as a Namsor gender probability score below 0.25. This removes 13,903 observations and 6,338 unique individuals. Next, we restrict the sample to core faculty, which we define as permanent faculty in the assistant professor, associate professor, and full professor ranks. This restriction excludes observations outside these main academic ranks, removes non-regular appointments such as visiting, adjunct, and emeritus positions, and also drops cases in which the reported rank is paired with unusually low experience. In particular, we exclude full professors with at most 6 years of experience and associate professors with at most 3 years of experience. This removes an additional 53,055 observations and 24,100 unique individuals. The resulting final sample contains 76,173 observations, 32,694 unique individuals, and 6,790 singleton observations.

We use the Namsor API for gender-assignment. The Namsor API returns a signed probability $p \in [-1, +1]$, with the sign encoding predicted gender (negative = male, positive = female) and the magnitude $|p|$ encoding confidence. We apply a confidence threshold of $|p| > 0.25$, treating observations with $|p| \leq 0.25$ as ambiguous and dropping them. Among the 63,132 pre-Namsor individuals, 6,338 (10.0%) fall below this threshold and 13,903 associated observations are dropped. In the final analytical sample of 32,694 individuals, the mean confidence is 0.920 for men and 0.874 for women, and the median confidence is 0.984 and 0.950, respectively. Table A1 reports the full distribution of $|p|$ in the final sample: 24,894 individuals (76.1%) have $|p| \geq 0.9$, and only 3,148 individuals (9.6%) have $|p| < 0.7$. To assess the reliability of the Namsor assignments, we additionally checked marginal names by hand, especially names of East-Asian and South-Asian origin where automated tools are known to be noisier. The robustness of the main results to a stricter $|p| \geq 0.9$ subsample is reported in Appendix C.9.

We examine singleton observations separately below. The final sample contains 6,790 singleton individuals (those observed in only one year), or 20.8% of the 32,694 individuals. Singletons are retained because the CRE specification conditions on individual means: a singleton's mean equals its single observation, so it contributes to the identification of time-invariant coefficients (such as *Female*) but not to within-individual identification of time-varying coefficients. The singleton share differs only marginally by gender (20.4% for men, 21.5% for women). Appendix C.7 reports a robustness check on the no-singletons and balanced subsamples.

Table A1. Distribution of Namsor confidence $|p|$ in the final analytical sample

Confidence bucket $\|p\|$	Male	Female	Total
< 0.70	1,957	1,191	3,148
0.70–0.80	1,027	816	1,843
0.80–0.90	980	1,829	2,809
0.90–0.95	771	1,586	2,357
0.95–0.99	11,472	5,244	16,716
≥ 0.99	5,812	9	5,821
Total	22,019	10,675	32,694

Notes: Distribution of Namsor confidence $|p|$ at the individual level in the final analytical sample (32,694 unique individuals). The signed Namsor probability $p \in [-1, +1]$ encodes predicted gender by sign (negative = male, positive = female) and confidence by magnitude.

B. Descriptive Statistics

Table B1 presents descriptive statistics for the full sample. Women account for 32% of the sample, consistent with persistent gender under-representation in academia. Salaries exhibit substantial right-skewness: the median faculty salary is $121,600, the mean salary is markedly higher at $143,617, reflecting a long upper tail driven by a relatively small group of very high-earning professors. The interquartile spread is also wide, indicating considerable heterogeneity in compensation.

Research output measures display even stronger dispersion. Faculty publish on average 39.9 papers, but the median is only 23, and the 75th percentile (50 publications) is five times the 25th percentile (10 publications). Log cumulative SJR scores show a similar pattern, with a mean of 38.14 versus a median of 19.31, and a large standard deviation of 60.13. Experience levels also vary meaningfully, with a median of 18 years since first publication and an interquartile range spanning from 11 to 28 years.

The rank distribution reflects a relatively balanced representation across positions, with 23% Assistant Professors, 29% Associate Professors, and 47% Full Professors. Field composition is driven primarily by Medical and Health Sciences (31%) and Natural Sciences (28%), followed by Social Sciences (24%). Agricultural Sciences, Engineering and Technology, and Humanities together account for less than 20% of the sample, underscoring the dominance of STEM-oriented disciplines.

Table B2 presents substantial variation in academic characteristics across fields, revealing both discipline-specific norms and broad structural patterns. Salaries vary widely, ranging from averages near $108,000 in the Humanities to above $162,000 in Medical and Health Sciences, with extreme upper tails in the latter. Research output also differs sharply by discipline: publication counts and cumulative SJR scores are modest in the Humanities and Social Sciences but exceedingly high in the Natural Sciences and Medical and Health Sciences, where the most productive scholars exceed 800–1,600 cumulative SJR points. Experience distributions further reflect field-specific career structures, with longer publication histories in the Natural Sciences and Agricultural Sciences, and shorter trajectories in the Humanities.

The proportion of women also differs markedly across fields, highlighting persistent patterns of occupational segregation. Representation is highest in the Humanities and Social Sciences, where women constitute roughly 40–44% of authors, and lowest in Engineering and Technology and in Economics-related subfields, where female representation ranges from 16–22%. Despite differences in magnitude, each field exhibits wide dispersion in salary and research output, indicating substantial heterogeneity in academic careers within disciplines.

Across all scientific domains examined, the descriptive statistics in Table B2 reveal systematic

gender gaps in key dimensions of academic careers, including salary, research output, and experience. These discrepancies are not confined to a single field; rather, they appear as a pervasive structural pattern affecting women across the entire spectrum of the academic workforce. The magnitude of these differences, paired with large and statistically significant t-statistics, suggests that they are not attributable to random variation but reflect deeply rooted and persistent inequalities.

In the Agricultural Sciences, women earn substantially lower salaries than men and exhibit markedly lower research productivity. Female academics publish, on average, 15 fewer articles than their male colleagues, produce roughly one-third less cumulative SJR-weighted output, and have over four fewer years of research experience. These gaps in experience, output, and reward already point to a cumulative disadvantage at early career stages, compounding over time through slower progression and reduced access to high-prestige collaborations.

Gender disparities intensify in Engineering and Technology, a field historically characterized by pronounced occupational segregation. Here, women earn nearly \$17,300 less than men despite facing comparable levels of salary dispersion, and their publication and cumulative SJR metrics lag considerably behind. The experience differential of nearly six years underscores a persistent underrepresentation of women, especially at senior levels.

In the Humanities, where overall publication expectations are lower, the gender gap remains pronounced but narrower in magnitude. Women publish fewer articles, accumulate substantially lower SJR scores, and report about 2.6 fewer years of research experience relative to their male peers. Even within fields traditionally perceived as more gender-balanced, the disparities suggest that structural and evaluative biases continue to shape academic trajectories.

The Medical and Health Sciences exhibit some of the largest discrepancies observed. Women earn, on average, almost \$40,000 less than men and show significant shortfalls in publication output and cumulative research impact. The differences in publication count (-22.8) and SJR score (-22.9) are among the largest across all fields, matched by an experience gap exceeding five years. A similar pattern emerges in the Natural Sciences, where women face both lower pay ($-\$13{,}759$) and substantially reduced research output. The publication and SJR gaps remain large (-19.5 and -20.1, respectively), accompanied by shorter research careers. The Social Sciences also show stark inequalities across all variables. Salary differences exceed \$31,000, and women publish significantly fewer articles and accumulate less than half the cumulative SJR score of men. Taken together, these descriptive patterns highlight a systemic and cross-disciplinary gender imbalance that persists even at advanced career stages.

Table B1. Descriptive Statistics

	Mean	Std. dev	Q25	Median	Q75
Female	0.32	0.47	0.00	0.00	1.00
Salary (in $)	143,617	88,473	90,557	121,600	174,100
Publication count	39.90	52.03	10.00	23.00	50.00
Log cumulative SJR score	38.14	60.13	6.27	19.31	45.85
Experience (in years)	19.80	11.47	11.00	18.00	28.00
Position:					
Assistant Professor	0.23	0.42	0.00	0.00	0.00
Associate Professor	0.29	0.45	0.00	0.00	1.00
Full Professor	0.47	0.50	0.00	0.00	1.00
Field:					
Agricultural Sciences	0.07	0.26	0.00	0.00	0.00
Engineering and Technology	0.06	0.25	0.00	0.00	0.00
Humanities	0.04	0.19	0.00	0.00	0.00
Medical and Health Sciences	0.31	0.46	0.00	0.00	1.00
Natural Sciences	0.28	0.45	0.00	0.00	1.00
Social Sciences	0.24	0.43	0.00	0.00	0.00

Notes: Experience (in years) is defined as the time elapsed since an author's first publication recorded in Scopus. The cumulative SJR score is weighted by the inverse of the square root of the number of co-authors for each publication.

Table B2. Descriptive Statistics by Field

	N	Mean	Std. dev	Min	Q25	Median	Q75	Max
Agricultural Sciences								
Female	5,355	0.30	0.46	0.00	0.00	0.00	1.00	1.00
Salary (in $)	5,355	121,219	45,504	20,655	91,333	114,216	143,096	500,001
Publication count	5,355	45.00	39.84	0.00	19.00	35.00	61.00	584.00
Cumulative SJR score	5,355	31.24	38.67	0.00	8.81	20.38	38.33	689.16
Experience	5,355	21.41	10.44	-1.00	13.00	21.00	29.00	54.00
Engineering and Technology								
Female	4,727	0.16	0.36	0.00	0.00	0.00	0.00	1.00
Salary (in $)	4,727	146,127	61,294	20,799	104,991	132,568	175,489	470,783
Publication count	4,727	49.39	55.84	0.00	17.00	33.00	61.00	709.00
Cumulative SJR score	4,727	40.06	56.38	0.00	10.58	23.25	47.79	661.83
Experience	4,727	20.72	11.58	-2.00	11.00	19.00	29.00	60.00
Humanities								
Female	2,773	0.44	0.50	0.00	0.00	0.00	1.00	1.00
Salary (in $)	2,773	108,453	43,770	20,606	78,600	97,340	127,327	320,257
Publication count	2,773	6.36	9.11	0.00	2.00	4.00	7.00	153.00
Cumulative SJR score	2,773	3.60	7.88	0.00	0.36	0.84	3.35	139.72
Experience	2,773	14.71	10.30	-2.00	7.00	12.00	20.00	56.00
Medical and Health Sciences								
Female	22,149	0.38	0.49	0.00	0.00	0.00	1.00	1.00
Salary (in $)	22,149	162,407	124,738	20,022	85,509	128,000	208,135	1,965,689
Publication count	22,149	45.22	56.40	0.00	11.00	26.00	58.00	862.00
Cumulative SJR score	22,149	38.19	59.55	0.00	5.66	18.22	46.02	1,004.48
Experience	22,149	19.43	11.51	0.00	10.00	18.00	27.00	68.00
Natural Sciences								
Female	20,352	0.20	0.40	0.00	0.00	0.00	0.00	1.00
Salary (in $)	20,352	137,560	62,260	20,069	96,554	123,686	165,113	804,701
Publication count	20,352	53.84	61.84	0.00	19.00	35.00	66.00	1,253.00
Cumulative SJR score	20,352	58.39	77.90	0.00	16.45	35.72	71.51	1,690.19
Experience	20,352	23.00	11.27	-1.00	14.00	22.00	31.00	66.00
Social Sciences								
Female	17,803	0.41	0.49	0.00	0.00	0.00	1.00	1.00
Salary (in $)	17,803	138,344	73,883	20,372	87,206	117,200	169,135	1,095,794
Publication count	17,803	19.40	24.79	0.00	6.00	12.00	23.00	465.00
Cumulative SJR score	17,803	22.92	35.05	0.00	3.86	10.59	26.99	497.03
Experience	17,803	17.11	11.15	-2.00	8.00	15.00	24.00	60.00

Notes: Experience (in years) is the time from the first Scopus-reported publication. The cumulative SJR score is discounted by the square root of the number of authors.

Table B3. Descriptive Statistics by Gender and Field

	Female		Male			
Variable	Mean	Std. Dev.	Mean	Std. Dev.	Difference	T-statistic
Agricultural Sciences						
Salary (in $)	116,651.89	44,300.21	123,219.06	45,884.82	−6,567.17	−4.94
Publication Count	34.58	29.91	49.56	42.69	−14.98	−14.71
Cumulative SJR score	23.76	26.48	34.52	42.52	−10.75	−11.24
Experience (in years)	18.48	9.65	22.69	10.51	−4.21	−14.29
Engineering and Technology						
Salary (in $)	131,558.10	55,600.30	148,822.01	61,922.54	−17,263.90	−7.61
Publication Count	36.36	52.18	51.79	56.17	−15.43	−7.29
Cumulative SJR score	27.58	45.56	42.36	57.87	−14.79	−7.74
Experience (in years)	15.79	10.36	21.63	11.57	−5.84	−13.81
Humanities						
Salary (in $)	104,202.68	40,843.98	111,796.49	45,676.20	−7,593.80	−4.61
Publication Count	4.98	6.38	7.43	10.66	−2.45	−7.51
Cumulative SJR score	2.38	4.66	4.57	9.58	−2.19	−7.88
Experience (in years)	13.23	9.10	15.86	11.02	−2.63	−6.89
Medical and Health Sciences						
Salary (in $)	137,983.76	89,596.41	177,426.64	139,999.19	−39,442.89	−25.56
Publication Count	31.08	39.25	53.92	63.17	−22.84	−33.19
Cumulative SJR score	24.03	38.91	46.90	67.80	−22.87	−31.89
Experience (in years)	16.22	10.12	21.40	11.86	−5.19	−34.66
Natural Sciences						
Salary (in $)	126,519.13	55,938.38	140,278.19	63,427.99	−13,759.06	−13.59
Publication Count	38.19	40.66	57.69	65.44	−19.50	−23.76
Cumulative SJR score	42.26	49.87	62.35	82.89	−20.09	−19.70
Experience (in years)	20.16	10.09	23.70	11.44	−3.54	−19.40
Social Sciences						
Salary (in $)	119,942.86	58,586.63	150,989.47	80,367.70	−31,046.61	−29.80
Publication Count	15.85	19.65	21.84	27.51	−6.00	−16.97
Cumulative SJR score	15.29	22.80	28.17	40.60	−12.88	−26.99
Experience (in years)	15.16	9.97	18.45	11.70	−3.28	−20.11

Notes: Experience (in years) is the time from the first Scopus-reported publication. The cumulative SJR score is discounted by the square root of the number of authors.

C. Robustness Checks

C.1. Gender Pay Gap and Gender Gap in Returns to Publications Across Fields

Significant differences in female-to-male ratios, compensation, and research productivity across academic fields can influence the size of the gender pay gap and the gap in returns to publications, as noted in previous sections. To begin with, we focus on the gender pay gap and show that it varies across the six broad academic fields (agricultural sciences, engineering and technology, humanities, medical and health sciences, natural sciences, social sciences), extending previous studies investigating gender disparities in pay.[4] For instance, prior work finds that a salary gap exists in the sciences. However, using the same survey data, others[5] report that salary differences between men and women in the humanities are not statistically significant.

Building upon these prior findings, we observe that although the gender pay gap varies by academic field, it remains negative and statistically significant when research productivity is not accounted for. Specifically, Column 1 of Table C1, which employs a pooled specification including only year and broad field fixed effects, reveals gender pay gaps disadvantageous to women ranging from 5.5% in agricultural sciences to 20.9% in social sciences. Humanities, natural sciences, engineering and technology, and medical and health sciences exhibit pay gaps of 6.4%, 9.6%, 12.3%, and 17.8%, respectively. Our findings align with those of prior work,[6] which documents that college-educated women frequently select academic majors linked to systematically lower wages compared to their male counterparts.

However, when productivity and its interaction with academic fields are introduced (Columns 3 and 4), the magnitude of the gap changes significantly in many fields. Notably, it becomes insignificant in agricultural sciences and humanities. Across the four pooled specifications, the R^2 does not exceed 21.5%, underscoring the need for models with better fit. Column 5 of Table C1 shows that the R^2 rises sharply to 85.8% under a correlated random effects (CRE) specification that accounts for persistent individual heterogeneity using individual-level controls constructed from Scopus IDs. Although the gender pay gap remains statistically significant for each field, its economic size changes compared to the pooled model without individual fixed effects. For instance, social sciences still exhibit the largest gap, 12.9%, yet individual productivity can mitigate that gap.

Finally, we investigate whether there is a gender gap in returns to publications in certain fields. Column 6 includes an interaction term between the female indicator and productivity, revealing substantial variation across fields. In medical and health sciences, female faculty members earn 10.7% less than their male counterparts for identical research output, rejecting the null hypothesis of no gender difference in conditional returns. Other fields show no statistically significant differences. Taken together, these results explain why no gender gap in returns to publications is detected in the aggregate sample across all disciplines, while also highlighting the importance of discipline-specific analyses for understanding the gender pay gap and the gender gap in returns to publications.

C.2. Gender Pay Gap Across Experience Groups

We further explore how the gender pay gap varies by rank, experience, and productivity.

Columns 1 and 2 of Table C2 reveal that women in lower academic ranks face a larger pay gap. Because Column 2 accounts for persistent individual heterogeneity through the CRE specification, it has substantially higher explanatory power, as indicated by R^2 of 85.7%. It shows that female assistant professors earn 7.7% less than their male counterparts, and female associate professors earn 4.7% less. Likewise, female full professors earn 2.9% less than male full professors, even though the proportion of women at senior ranks is substantially smaller than at junior levels. A decreasing gender gap for more senior faculty is in line with previous evidence,[7] which shows that women perform better in terms of salary progression after promotion. These heterogeneity patterns are also consistent with the literature on parenthood penalties in academia. The larger gender gaps

Table C1. Gender Pay Gap Across Fields

	Pooled (1)	Pooled (2)	Pooled (3)	Pooled (4)	CRE (5)	CRE (6)
Female ×						
Agricultural Sciences	-0.055^{***}	-0.097^{***}	-0.016	-0.018	-0.022	-0.068
	(0.011)	(0.011)	(0.010)	(0.027)	(0.020)	(0.050)
Engineering and Technology	-0.123^{***}	-0.100^{***}	-0.037^{**}	-0.104^{**}	-0.064^{*}	-0.139^{*}
	(0.016)	(0.016)	(0.014)	(0.038)	(0.028)	(0.066)
Humanities	-0.064^{***}	-0.101^{***}	-0.012	-0.012	0.024	0.060
	(0.014)	(0.013)	(0.013)	(0.017)	(0.023)	(0.031)
Medical and Health Sciences	-0.178^{***}	-0.119^{***}	-0.023^{**}	0.065^{***}	-0.053^{***}	0.042^{*}
	(0.009)	(0.008)	(0.008)	(0.018)	(0.009)	(0.019)
Natural Sciences	-0.096^{***}	-0.087^{***}	-0.037^{***}	-0.165^{***}	-0.069^{***}	-0.200^{***}
	(0.007)	(0.007)	(0.006)	(0.021)	(0.012)	(0.035)
Social Sciences	-0.209^{***}	-0.109^{***}	-0.055^{***}	-0.057^{***}	-0.129^{***}	-0.113^{***}
	(0.007)	(0.006)	(0.006)	(0.012)	(0.010)	(0.020)
Productivity ×						
Agricultural Sciences			0.167^{***}	0.168^{***}	0.120^{***}	0.068
			(0.003)	(0.004)	(0.035)	(0.046)
Engineering and Technology			0.183^{***}	0.182^{***}	0.158^{***}	0.152^{***}
			(0.003)	(0.003)	(0.033)	(0.037)
Humanities			0.176^{***}	0.177^{***}	0.102	0.179^{*}
			(0.008)	(0.009)	(0.060)	(0.082)
Medical and Health Sciences			0.175^{***}	0.181^{***}	0.328^{***}	0.378^{***}
			(0.003)	(0.003)	(0.017)	(0.022)
Natural Sciences			0.173^{***}	0.173^{***}	0.135^{***}	0.116^{***}
			(0.002)	(0.002)	(0.021)	(0.024)
Social Sciences			0.178^{***}	0.179^{***}	0.084^{***}	0.069^{***}
			(0.002)	(0.003)	(0.014)	(0.019)
Female × *Productivity* ×						
Agricultural Sciences				0.001		0.124
				(0.010)		(0.069)
Engineering and Technology				0.025^{*}		0.029
				(0.013)		(0.082)
Humanities				0.002		-0.165
				(0.017)		(0.120)
Medical and Health Sciences				-0.035^{***}		-0.107^{***}
				(0.006)		(0.031)
Natural Sciences				0.040^{***}		0.083
				(0.006)		(0.048)
Social Sciences				0.002		0.033
				(0.005)		(0.028)
Num. Obs.	76,089	76,089	76,089	76,089	76,089	76,089
R^2	0.032	0.098	0.214	0.215	0.858	0.858
FE: Year	X	X	X	X	X	X
FE: Broad Field	X					
FE: Narrow Field		X	X	X	X	X
FE: Scopus ID					X	X

Notes: + $p < 0.1$, * $p < 0.05$, ** $p < 0.01$, *** $p < 0.001$. Standard errors in parentheses.

observed among junior faculty and the gradual narrowing of gaps with experience may reflect the disproportionate effect of family-related career interruptions and caregiving responsibilities during early-career stages. Because our data do not directly observe parenthood status or career breaks, we cannot test this mechanism directly, but the observed patterns are broadly consistent with prior evidence in the literature.

Intriguingly, results in Column 6 suggest that controlling for individual characteristics, the gender pay gap diminishes progressively with faculty experience and eventually vanishes for faculty with more than 35 years in academia, implying that discrimination is more pronounced early in academic careers.

Next, we perform an analogous sensitivity analysis of returns to publications. To assess the role of productivity, we divide the sample into halves based on SJR scores. H1 denotes scholars in the top half of the productivity distribution (higher productivity), while H2 denotes scholars in the bottom half of the productivity distribution (lower productivity). Column 2 shows significant returns for assistant and associate professors, yet none for full professors, potentially reflecting career-family trade-offs or movement into non-academic opportunities for the most productive scholars. Columns 3 and 4 of Table C2 indicate that the largest pay gaps in returns to publications of 9.4% and 8.9%, respectively, without and with controls for individual heterogeneity, occur among the most productive half of researchers. The analysis controls for narrow research fields with fixed effects for each of nearly 300 different sub-disciplines and individual characteristics, so at least part of the gap may reflect discriminatory mechanisms. Finally, Column 6 controls for individual characteristics and indicates that returns to productivity grow during the first 20 years of experience but turn negative after 35 years, which could stem from age-related discrimination or other factors.

Table C2. Gender Pay Gap Across Experience Groups

	Pooled (1)	CRE (2)	Pooled (3)	CRE (4)	Pooled (5)	CRE (6)
Female × Assistant Professor	−0.055***	−0.077***				
	(0.007)	(0.010)				
Female × Associate Professor	−0.017**	−0.047***				
	(0.006)	(0.010)				
Female × Full Professor	−0.005	−0.029**				
	(0.006)	(0.009)				
Productivity × Assistant Prof.	0.049***	0.177***				
	(0.003)	(0.011)				
Productivity × Associate Prof.	0.080***	0.174***				
	(0.003)	(0.023)				
Productivity × Full Professor	0.115***	−0.041				
	(0.002)	(0.031)				
Female × Productivity H1			−0.094***	−0.089***		
			(0.014)	(0.009)		
Female × Productivity H2			0.011	−0.049***		
			(0.023)	(0.010)		
Productivity × H1			0.101***	0.174***		
			(0.004)	(0.012)		
Productivity × H2			0.213***	0.109***		
			(0.004)	(0.028)		
Female × Experience < 5 Y					−0.042***	−0.073***
					(0.011)	(0.016)
Female × Experience 5–9 Y					−0.052***	−0.083***
					(0.009)	(0.013)
Female × Experience 10–14 Y					−0.007	−0.036*
					(0.009)	(0.014)
Female × Experience 15–19 Y					−0.041***	−0.071***
					(0.009)	(0.015)
Female × Experience 20–24 Y					−0.016	−0.046**
					(0.011)	(0.017)
Female × Experience 25–29 Y					−0.031*	−0.063***
					(0.012)	(0.019)
Female × Experience 30–34 Y					−0.012	−0.051*
					(0.015)	(0.022)
Female × Experience 35–39 Y					−0.037	−0.051
					(0.021)	(0.031)
Female × Experience > 40 Y					0.075**	0.047
					(0.026)	(0.039)
Productivity × Exp. < 5 Y					0.098***	0.141***
					(0.006)	(0.013)
Productivity × Exp. 5–10 Y					0.076***	0.216***
					(0.004)	(0.018)
Productivity × Exp. 10–15 Y					0.104***	0.201***
					(0.004)	(0.025)
Productivity × Exp. 15–20 Y					0.130***	0.194***
					(0.004)	(0.035)
Productivity × Exp. 20–25 Y					0.143***	0.063
					(0.005)	(0.050)
Productivity × Exp. 25–30 Y					0.155***	−0.017
					(0.006)	(0.064)
Productivity × Exp. 30–35 Y					0.173***	−0.145
					(0.006)	(0.085)
Productivity × Exp. 35–40 Y					0.174***	−0.545***
					(0.008)	(0.129)
Productivity × Exp. > 40 Y					0.197***	−1.145***
					(0.009)	(0.186)
Num. obs.	76,173	76,173	76,173	76,173	76,173	76,173
R^2	0.286	0.857	0.227	0.858	0.235	0.858
FE: Position	X	X				
FE: Top/Bottom Half			X	X		
FE: Experience Group					X	X
FE: Narrow Field	X	X	X	X	X	X
FE: Scopus ID		X		X		X
FE: Year	X	X	X	X	X	X

Notes: + $p < 0.1$, * $p < 0.05$, ** $p < 0.01$, *** $p < 0.001$. Standard errors in parentheses.

C.3. Gender Pay Gap Across Lags

Because academic research may not translate into monetary rewards immediately, we construct robustness checks using lagged SJR performance measures. Table C3 shows that the results remain qualitatively unchanged, with no visible gender gap in returns to productivity. These results hold consistently for lag structures of one, two, and three years. Furthermore, the coefficient on productivity diminishes with longer lags, suggesting that salary rewards tend to materialize within the first few years following publication.

Table C3. Gender Pay Gap Across Lags

	Pooled (1)	CRE (2)	Pooled (3)	CRE (4)	Pooled (5)	CRE (6)
Female	-0.025^{**}	-0.058^{***}	-0.026^{**}	-0.059^{***}	-0.028^{***}	-0.062^{***}
	(0.009)	(0.013)	(0.009)	(0.012)	(0.008)	(0.012)
Productivity (Lag 1)	0.174^{***}	0.133^{***}				
	(0.002)	(0.011)				
Female × Productivity (Lag 1)	−0.003	0.006				
	(0.003)	(0.015)				
Productivity (Lag 2)			0.171^{***}	0.100^{***}		
			(0.002)	(0.010)		
Female × Productivity (Lag 2)			−0.003	−0.003		
			(0.003)	(0.014)		
Productivity (Lag 3)					0.168^{***}	0.102^{***}
					(0.002)	(0.009)
Female × Productivity (Lag 3)					−0.002	0.001
					(0.003)	(0.014)
Num. Obs.	76,173	76,173	76,173	76,173	76,173	76,173
Adj. R^2	0.216	0.857	0.218	0.857	0.221	0.857
FE: Year	X	X	X	X	X	X
FE: Broad Field	X	X	X	X	X	X

Notes: $+\ p < 0.1$, $^{*}\ p < 0.05$, $^{**}\ p < 0.01$, $^{***}\ p < 0.001$. Standard errors in parentheses.

C.4. Robustness to Co-author Weighting Scheme

Table C4 replicates the Panel A specification of Table 5 (solo versus co-authored decomposition) under three alternative weighting schemes for co-authored productivity: *unweighted* (full credit, no adjustment for team size), $1/n$ (equal credit), and the baseline $1/\sqrt{n}$. Solo productivity is identical across schemes by construction, since $n = 1$ for solo papers. The Female × Productivity (solo-authored) interaction is positive, statistically significant, and economically similar across all three schemes (5.6%–6.2% in the CRE specification with interactions). The Female × Productivity (co-authored) interaction remains statistically insignificant under each weighting scheme. We conclude that the credit-attribution results are not driven by the particular co-authorship-weighting convention.

Table C4. Table 5 Panel A under alternative co-author weighting schemes

	Unweighted		$1/n$		$1/\sqrt{n}$ (baseline)	
	Pooled+Int.	CRE+Int.	Pooled+Int.	CRE+Int.	Pooled+Int.	CRE+Int.
Female	-0.020^{**}	-0.056^{***}	-0.032^{***}	-0.056^{***}	-0.025^{***}	-0.054^{***}
	(0.008)	(0.011)	(0.007)	(0.010)	(0.007)	(0.011)
Productivity (co-authored)	0.073^{***}	0.134^{***}	0.098^{***}	0.150^{***}	0.085^{***}	0.148^{***}
	(0.002)	(0.010)	(0.002)	(0.014)	(0.002)	(0.012)
Productivity (solo-authored)	0.022^{***}	-0.018	0.012^{***}	-0.017	0.017^{***}	-0.018
	(0.002)	(0.014)	(0.002)	(0.014)	(0.002)	(0.014)
Female × Prod. (co-authored)	-0.010^{***}	-0.007	-0.010^{***}	0.027	-0.010^{***}	0.009
	(0.002)	(0.014)	(0.002)	(0.021)	(0.002)	(0.017)
Female × Prod. (solo-authored)	0.014^{***}	0.062^{**}	0.016^{***}	0.056^{*}	0.015^{***}	0.059^{*}
	(0.004)	(0.023)	(0.004)	(0.023)	(0.004)	(0.023)
Num. obs.	76,173	76,173	76,173	76,173	76,173	76,173
R^2	0.404		0.407		0.405	
R^2 (cond.)		0.858		0.858		0.858
FE: Year	X	X	X	X	X	X
FE: Experience-Year Bins	X	X	X	X	X	X
FE: University	X	X	X	X	X	X
FE: Narrow Field	X	X	X	X	X	X
FE: Scopus ID		X		X		X

Notes: + $p < 0.1$, * $p < 0.05$, ** $p < 0.01$, *** $p < 0.001$. Robustness check on Panel A of Table 5: each pair of columns re-estimates the solo/co-authored decomposition under a different weighting of co-authored productivity. "Unweighted" uses full credit (no adjustment for team size); "$1/n$" assigns equal credit; "$1/\sqrt{n}$" is the baseline modified-fractional scheme. Solo productivity is identical across columns by construction. Heteroskedasticity-robust standard errors in parentheses.

C.5. Co-author Team Size and Author Position

Table C5 reports paper-level descriptive statistics on co-authorship patterns by gender and broad field: the number of papers, the share of solo-authored papers, the share of papers in which the focal author appears as first author, the share in which they appear as last author, and the mean number of co-authors per paper.

Table C5. Co-author team size and author-position shares, by broad field and gender

Field	Gender	N papers	Share solo	Share first	Share last	Mean team
All fields						
	Men	112,735	0.025	0.094	0.356	7.93
	Women	40,353	0.034	0.148	0.302	7.81
Agricultural Sciences	Men	10,583	0.011	0.091	0.345	6.94
	Women	3,796	0.007	0.117	0.364	6.65
Engineering and Technology	Men	11,565	0.010	0.052	0.520	5.01
	Women	1,854	0.008	0.076	0.499	5.24
Humanities	Men	660	0.547	0.138	0.192	2.26
	Women	405	0.570	0.131	0.158	2.44
Medical and Health Sciences	Men	36,248	0.009	0.085	0.283	9.99
	Women	17,804	0.009	0.142	0.251	9.49
Natural Sciences	Men	41,236	0.018	0.069	0.387	8.53
	Women	8,343	0.014	0.087	0.363	9.38
Social Sciences	Men	12,391	0.098	0.241	0.331	3.80
	Women	8,138	0.102	0.258	0.285	3.90
Other	Men	52	0.039	0.019	0.365	6.04
	Women	13	0.000	0.077	0.231	7.15

Notes: Paper-level descriptive statistics for the analytical sample. *N papers* is the total number of (author–paper) pairs in each subgroup. *Share solo*, *share first*, and *share last* report the fraction of papers in which the focal author is the sole, first, or last author, respectively. *Mean team* is the average number of co-authors per paper. Field assignment is at the paper level using the SJR broad-field classification of the publishing journal. "Other" collects a small residual of papers in journals without an SJR broad-field classification.

C.6. Robustness to Explicit Rank Controls

Table C6 re-estimates Columns 3–6 of Table 2 after adding explicit academic-rank indicators (assistant / associate / full professor) to the specification. The main coefficients are qualitatively unchanged. Table C7 additionally allows the rank–salary relationship to vary by year, replacing the constant rank dummies with their full interaction with year. The coefficients are essentially identical, indicating that the rank–salary mapping is stable within our short 2017–2019 panel.

Table C6. Table 2, Columns 3–6 with explicit rank controls added

	Pooled		CRE	
	(3′) +Rank	(4′) +Rank	(5′) +Rank	(6′) +Rank
Female	-0.039^{***}	-0.028^{***}	-0.058^{***}	-0.067^{***}
	(0.003)	(0.008)	(0.005)	(0.011)
Productivity	0.066^{***}	0.067^{***}	0.159^{***}	0.155^{***}
	(0.002)	(0.002)	(0.010)	(0.012)
Female × Productivity		-0.004		0.010
		(0.003)		(0.017)
Num. obs.	76,173	76,173	76,173	76,173
R^2	0.449	0.449		
R^2 (cond.)			0.857	0.857
FE: Year	X	X	X	X
FE: Experience-Year Bins	X	X	X	X
FE: University	X	X	X	X
FE: Narrow Field	X	X	X	X
FE: Rank	X	X	X	X
FE: Scopus ID			X	X

Notes: + $p < 0.1$, * $p < 0.05$, ** $p < 0.01$, *** $p < 0.001$. Heteroskedasticity-robust standard errors in parentheses. Rank indicators are dummies for assistant, associate, and full professor. Columns mirror Columns 3–6 of Table 2, with rank added as an additional control (in the CRE columns, rank dummies are included via the CRE demeaning scheme).

Table C7. Rank as a constant control vs. Rank × Year interactions (pooled OLS)

	Rank (constant)<br>(1)	Rank × Year<br>(2)
Female	-0.028^{*}	-0.028^{*}
	(0.011)	(0.011)
Productivity	0.067^{***}	0.067^{***}
	(0.003)	(0.003)
Female × Productivity	-0.004	-0.004
	(0.004)	(0.004)
Num. obs.	76,173	76,173
R^2 (full)	0.449	0.450
FE: Year	X	X
FE: Narrow field	X	X
FE: University	X	X
FE: Experience-year bins	X	X
FE: Rank	X	
FE: Rank × Year		X

Notes: + $p < 0.1$, * $p < 0.05$, ** $p < 0.01$, *** $p < 0.001$. Pooled OLS with heteroskedasticity-robust standard errors clustered at the individual (Scopus ID) level. Column (1) replicates Column (4) of the rank-robustness specification (rank entered as a constant control alongside experience-year bins). Column (2) replaces the constant rank dummies with their full interaction with year, allowing the rank–salary mapping to vary over time. The main coefficients are unchanged, indicating that the rank–salary relationship is stable within our short 2017–2019 panel.

C.7. Robustness to Singleton and Balanced Subsamples

Tables C8 and C9 re-estimate the Table 2 specifications on (a) the subsample excluding singletons ($N = 69{,}383$) and (b) the balanced subsample of individuals observed in all three years ($N = 52{,}725$). The main coefficients are qualitatively unchanged in both subsamples.

Table C8. Table 2 on the no-singletons subsample

	Pooled				CRE	
	(1)	(2)	(3)	(4)	(5)	(6)
Female	-0.156^{***}	-0.061^{***}	-0.040^{***}	-0.018^{*}	-0.062^{***}	-0.052^{***}
	(0.004)	(0.004)	(0.004)	(0.008)	(0.005)	(0.013)
Productivity		0.141^{***}	0.092^{***}	0.095^{***}	0.169^{***}	0.165^{***}
		(0.002)	(0.002)	(0.002)	(0.010)	(0.012)
Female × Productivity				-0.008^{**}		0.010
				(0.003)		(0.017)
Num. obs.	69,383	69,383	69,383	69,383	69,383	69,383
FE: Year	X	X	X	X	X	X
FE: Experience-Year Bins			X	X	X	X
FE: University			X	X	X	X
FE: Narrow Field			X	X	X	X
FE: Scopus ID					X	X

Notes: + $p < 0.1$, * $p < 0.05$, ** $p < 0.01$, *** $p < 0.001$. Subsample excludes individuals observed in only one year (N = 69,383 observations from 25,904 individuals). Heteroskedasticity-robust standard errors in parentheses.

Table C9. Table 2 on the balanced subsample

	Pooled				CRE	
	(1)	(2)	(3)	(4)	(5)	(6)
Female	-0.155^{***}	-0.056^{***}	-0.039^{***}	-0.020^{*}	-0.060^{***}	-0.059^{***}
	(0.005)	(0.005)	(0.004)	(0.010)	(0.007)	(0.015)
Productivity		0.144^{***}	0.095^{***}	0.097^{***}	0.168^{***}	0.165^{***}
		(0.002)	(0.002)	(0.003)	(0.011)	(0.013)
Female × Productivity				-0.007^{*}		0.009
				(0.003)		(0.019)
Num. obs.	52,725	52,725	52,725	52,725	52,725	52,725
FE: Year	X	X	X	X	X	X
FE: Experience-Year Bins			X	X	X	X
FE: University			X	X	X	X
FE: Narrow Field			X	X	X	X
FE: Scopus ID					X	X

Notes: + $p < 0.1$, * $p < 0.05$, ** $p < 0.01$, *** $p < 0.001$. Balanced subsample restricted to individuals observed in all three years (N = 52,725 observations from 17,575 individuals). Heteroskedasticity-robust standard errors in parentheses.

C.8. Robustness to University-Level Clustering

Table C10 replicates Table 2 with standard errors clustered at the university (OpenTheBooks ID) level rather than at the individual (Scopus ID) level used in the main text. The CRE columns drop 28 individuals observed at more than one university, since cluster-robust variance estimation requires individuals to be nested within a single cluster. The main coefficients and their statistical significance are unchanged.

Table C10. Table 2 with standard errors clustered at the university (OpenTheBooks ID) level

	Pooled				CRE	
	(1)	(2)	(3)	(4)	(5)	(6)
Female	-0.154^{***}	-0.060^{***}	-0.041^{***}	-0.017	-0.062^{***}	-0.049^{**}
	(0.011)	(0.011)	(0.008)	(0.012)	(0.010)	(0.017)
Productivity		0.138^{***}	0.091^{***}	0.094^{***}	0.170^{***}	0.165^{***}
		(0.009)	(0.010)	(0.010)	(0.041)	(0.047)
Female × Productivity				-0.009^{**}		0.010
				(0.003)		(0.031)
Num. obs.	76173	76173	76173	76173	76100	76100
R^2	0.019	0.126	0.405	0.405	0.858	0.858
FE: Year	*X*	*X*	*X*	*X*	*X*	*X*
FE: Experience Years Bins			*X*	*X*	*X*	*X*
FE: University ID			*X*	*X*	*X*	*X*
FE: Narrow Field			*X*	*X*	*X*	*X*
FE: Scopus ID					*X*	*X*

Notes: + $p < 0.1$, * $p < 0.05$, ** $p < 0.01$, *** $p < 0.001$. Standard errors clustered at the university (OpenTheBooks ID) level. The CRE columns exclude 28 individuals observed at more than one university during the panel period, since cluster-robust variance estimation requires individuals to be nested within a single cluster.

C.9. Robustness to High-Confidence Gender Assignment

Table C11 replicates the Table 2 specifications on the subsample restricted to individuals with high-confidence Namsor gender assignments ($|p| \geq 0.9$, covering 76.1% of the analytical sample, or 58,099 observations from 24,894 individuals). The estimated coefficients are virtually identical to those in Table 2, confirming that ambiguous gender assignments have no material impact on the main findings.

Table C11. Table 2 on the high-confidence Namsor subsample ($|p| \geq 0.9$)

	Pooled				CRE	
	(1)	(2)	(3)	(4)	(5)	(6)
Female	-0.165^{***}	-0.069^{***}	-0.047^{***}	-0.020^{*}	-0.068^{***}	-0.049^{***}
	(0.005)	(0.005)	(0.004)	(0.010)	(0.006)	(0.014)
Productivity		0.138^{***}	0.094^{***}	0.097^{***}	0.179^{***}	0.185^{***}
		(0.002)	(0.002)	(0.002)	(0.012)	(0.015)
Female × Productivity				-0.010^{**}		-0.016
				(0.003)		(0.021)
Num. obs.	58,099	58,099	58,099	58,099	58,099	58,099
R^2	0.020	0.128	0.404	0.404	0.856	0.856
FE: Year	X	X	X	X	X	X
FE: Experience-Year Bins			X	X	X	X
FE: University			X	X	X	X
FE: Narrow Field			X	X	X	X
FE: Scopus ID					X	X

Notes: + $p < 0.1$, * $p < 0.05$, ** $p < 0.01$, *** $p < 0.001$. Sample restricted to individuals with high-confidence Namsor gender assignments ($|p| \geq 0.9$), covering 24,894 individuals (76.1% of the analytical sample) and 58,099 observations. Heteroskedasticity-robust standard errors in parentheses.

D. Matching and Weighting

Tables D1–D3 present the balance and robustness results for the matching and weighting estimators. Table D1 reports the balance of covariates after coarsened exact matching (CEM), nearest neighbour (NN), nearest neighbour with replacement (NNR), and inverse probability weighting (IPW). Table D2 repeats the main regression specification using these matched and weighted samples, additionally including salary in the first observed year as a matching variable. Table D3 presents the corresponding balance diagnostics for the specification including salary.

Table D1. Balancing after Matching and Weighting

	CEM		NN		NNR		IPW	
	Female	Male	Female	Male	Female	Male	Female	Male
Log cumulative SJR score	2.356	2.360	2.343	2.395	2.343	2.347	2.343	2.335
Experience (in years)	15.475	15.463	15.592	15.742	15.592	15.482	15.592	15.473
Position:								
Assistant Professor	0.331	0.331	0.331	0.326	0.331	0.333	0.331	0.334
Associate Professor	0.326	0.326	0.326	0.325	0.326	0.324	0.326	0.326
Full Professor	0.343	0.343	0.342	0.349	0.342	0.342	0.342	0.340
Field:								
Agricultural Sciences	0.064	0.064	0.068	0.068	0.068	0.066	0.068	0.068
Engineering and Technology	0.030	0.030	0.032	0.032	0.032	0.033	0.032	0.032
Humanities	0.046	0.046	0.050	0.052	0.050	0.048	0.050	0.050
Medical and Health Sciences	0.369	0.369	0.365	0.361	0.365	0.368	0.365	0.364
Natural Sciences	0.173	0.173	0.171	0.172	0.171	0.171	0.171	0.172
Social Sciences	0.318	0.318	0.314	0.315	0.314	0.315	0.314	0.314

Notes: Experience (in years) is the time from the first Scopus-reported publication. Matching methods: CEM (Coarsened Exact Matching), NN (Nearest Neighbour), NNR (Nearest Neighbour with Replacement), IPW (Inverse Probability Weighting).

Table D2. Robustness: Matching and Weighting with salary as a matching variable

Variable	CEM (1)	NN (2)	NNR (3)	IPW (4)
Female	-0.035^{**}	-0.077^{***}	-0.073^{***}	-0.062^{***}
	(0.012)	(0.013)	(0.015)	(0.012)
Productivity	0.147^{***}	0.158^{***}	0.129^{***}	0.186^{***}
	(0.012)	(0.015)	(0.016)	(0.011)
Female × Productivity	−0.014	−0.029	−0.011	-0.057^{***}
	(0.017)	(0.019)	(0.020)	(0.015)
Num. obs.	53,652	49,381	41,204	76,089
R^2	0.827	0.850	0.845	0.893
FE: Scopus ID	X	X	X	X
FE: Year	X	X	X	X
FE: University	X	X	X	X

Notes: $+\ p < 0.1$, $^{*}\ p < 0.05$, $^{**}\ p < 0.01$, $^{***}\ p < 0.001$. Standard errors in parentheses. All regression results obtained using a CRE model.

Table D3. Balancing after Matching and Weighting including Salary

	CEM		NN		NNR		IPW	
	Female	Male	Female	Male	Female	Male	Female	Male
Log salary at $t = 1$	11.611	11.611	11.582	11.600	11.582	11.584	11.582	11.576
Log cumulative SJR score	2.421	2.424	2.343	2.407	2.343	2.337	2.343	2.336
Experience (in years)	15.395	15.375	15.592	15.780	15.592	15.399	15.592	15.489
Position:								
Assistant Professor	0.336	0.336	0.331	0.325	0.331	0.334	0.331	0.333
Associate Professor	0.324	0.324	0.326	0.332	0.326	0.329	0.326	0.326
Full Professor	0.340	0.340	0.342	0.342	0.342	0.337	0.342	0.340
Field:								
Agricultural Sciences	0.057	0.057	0.068	0.071	0.068	0.070	0.068	0.068
Engineering and Technology	0.027	0.027	0.032	0.032	0.032	0.033	0.032	0.032
Humanities	0.043	0.043	0.050	0.051	0.050	0.049	0.050	0.050
Medical and Health Sciences	0.359	0.359	0.365	0.363	0.365	0.362	0.365	0.366
Natural Sciences	0.187	0.187	0.171	0.173	0.171	0.175	0.171	0.172
Social Sciences	0.327	0.327	0.314	0.310	0.314	0.311	0.314	0.313

Notes: Experience (in years) is the time from the first Scopus-reported publication. Matching methods: CEM (Coarsened Exact Matching), NN (Nearest Neighbour), NNR (Nearest Neighbour with Replacement), IPW (Inverse Probability Weighting). The matching here additionally includes log salary in the first observed year as a balancing variable.

References

[1] Quacquarelli Symonds (QS). QS World University Rankings 2020. `https://www.topuniversities.com/university-rankings/world-university-rankings/2020`, 2020. Accessed 2021-03-22.

[2] Times Higher Education. Wall Street Journal/Times Higher Education: United States University Rankings (2020 edition). `https://www.timeshighereducation.com/rankings/united-states/2020`, 2020. Accessed 2021-03-22.

[3] American Transparency. OpenTheBooks.com: FAQs / About the database. `https://www.openthebooks.com/faq/`, 2021. Accessed 2021-03-22.

[4] Donna K Ginther. Does science discriminate against women? EVIDENCE from academia, 1973-1997. *FRB Atlanta Working Paper*, 2001.

[5] Donna K Ginther and Kathy J Hayes. Gender differences in salary and promotion for faculty in the humanities 1977–95. *Journal of Human Resources*, 38(1):34–73, 2003.

[6] Carolyn M. Sloane, Erik G. Hurst, and Dan A. Black. College majors, occupations, and the gender wage gap. *Journal of Economic Perspectives*, 35(4):223–48, November 2021. doi: 10.1257/jep.35.4.223. URL `https://www.aeaweb.org/articles?id=10.1257/jep.35.4.223`.

[7] Laura Hospido, Luc Laeven, and Ana Lamo. The gender promotion gap: Evidence from central banking. *Review of Economics and Statistics*, 104(5):981–996, 2022.
